# Effects of acute slackline exercise on executive function in college students

**DOI:** 10.3389/fpsyg.2023.1092804

**Published:** 2023-04-20

**Authors:** Ching-Tsai Wen, Chiung-Ling Chu, Hsueh-Chih Chen, Ting-Yu Chueh, Chih-Chien Lin, Shao-Yu Wu, Wei-Chen Hsu, Chung-Ju Huang, Tsung-Min Hung

**Affiliations:** ^1^Department of Sport and Leisure, National Quemoy University, Kinmen, Taiwan; ^2^Department of Physical Education and Sport Sciences, National Taiwan Normal University, Taipei, Taiwan; ^3^Department of Educational Psychology and Counseling, National Taiwan Normal University, Taipei, Taiwan; ^4^Department of Kinesiology, University of Maryland, College Park, MD, United States; ^5^Graduate Institute of Sport Pedagogy, University of Taipei, Taipei, Taiwan; ^6^Institute for Research Excellence in Learning Science, National Taiwan Normal University, Taipei, Taiwan

**Keywords:** executive functions, exercise, slackline, college students, acute

## Abstract

**Background:**

Physical exercise as an intervention for improving cognitive function, especially executive function, is receiving increasing attention because it is easily accessible, cost-effective and promises many additional health-related benefits. While previous studies focused on aerobic exercise and resistance exercise, recent findings have suggested that exercise with high coordination demand elicits beneficial effects on executive function. We therefore examined the effects of an acute slackline exercise on the executive functions of young adults.

**Methods:**

In a crossover experimental design, 47 healthy participants (21 females), ranging in age from 18 to 27 years (*M* = 19.17, SD = 1.94) were randomly assigned to different sequences of two conditions (slackline exercise and film-watching). Before and after the 50 min intervention, a modified Simon task was used to assess participants’ executive function (inhibitory control and cognitive flexibility).

**Results:**

College students showed better inhibitory control performance as indicated by shorter reaction times following acute slackline exercise than those who participated in the film-watching session. As there was no difference in accuracy between the slackline exercise and film-watching sessions, the shortened reaction time after slackline exercise provides evidence against a simple speed-accuracy trade-off.

**Conclusion:**

Compared with film-watching, acute slackline exercise provides favorable effects on executive function necessitating inhibition in young adults. These findings provide insight into exercise prescription and cognition, and further evidence for the beneficial effects of coordination exercise on executive functions.

## Introduction

The executive functions refer to the family of top-down mental processes that are essential for achieving internal goals and orchestrating thoughts and plans (Diamond, 2013). Executive functions include three fundamental processes that underpin goal-directed behaviors: inhibition, working memory, and cognitive flexibility. Inhibition involves being able to control one’s attention, behavior, thoughts, and/or emotions to override a strong internal predisposition or external lure, and instead do what is more appropriate or needed (Miyake et al., 2000; Diamond, 2013). Working memory is a psychological construct for the temporary storage and manipulation of information required to perform intricate cognitive tasks such as reasoning and decision-making (Diamond, 2013). Cognitive flexibility, also called set shifting, mental flexibility, or mental set shifting, is the ability to switch between modes of thought and to simultaneously think about multiple concepts (Diamond, 2013), such as thinking out multiple solutions to a problem, switching freely between different categories of knowledge, and inhibiting interferential prepotent responses to achieve a specific goal (Rende, 2000; Johnco et al., 2014). These cognitive abilities are important in ensuring appropriate behavior and decision making, as well as for maintaining mental and physical health (Diamond, 2013), and they have been proposed to underlie socioemotional development and academic skills (Bull et al., 2011; Diamond, 2013).

A growing body of research has investigated the relationship between physical activity and executive function with an eye toward understanding how sedentary behavior might negatively impact not only physical health, but cognitive health. The Scientific Report of the Physical Activity Guidelines Advisory Committee (2018) highlighted the importance of physical activity for sustaining optimal levels of brain health. Emerging evidence suggests that chronic physical activity engagement plays a pivotal role in enhancing executive function (Voelcker-Rehage and Niemann, 2013; Vazou et al., 2016; Ludyga et al., 2020). Acute bouts of physical activity have also been shown to result in facilitating effects on executive function (Ludyga et al., 2016; Moreau and Chou, 2019; Haverkamp et al., 2020; Chueh et al., 2022). That is, participating in acute exercise can not only reduce the time of sedentary behavior but also generate a transient improvement in executive functions. Although studies have mainly focused on the effects of aerobic and resistance exercise on executive function, finding other exercise types to improve executive function can provide more options.

Theoretical hypotheses postulate that exercises involving high motor skills and coordination demands may enhance executive functions because of the pre-activation of motor-related brain regions such as the prefrontal cortex and supplementary motor areas serving executive function (Best, 2010; Diamond and Lee, 2011). The positive effects of acute coordination exercises on executive functions of older children and adolescents have been shown (Budde et al., 2008; Jäger et al., 2014). Previous studies designed exercise contexts that may also demand aerobic-related components. For example, Stein et al. (2017) designed an exercise intervention including various combinations of jumping and running activities, which largely necessitated aerobic energy systems. It is difficult to separate the impact of acute coordination exercise on executive functions from the impact of aerobic exercise on executive functions in these studies. Thus, this study aimed to examine the acute effects of physical activity that predominantly targets motor skills and coordination components on executive functions in young adults.

Slacklining is a novel recreational activity that has gained popularity in recent years among young adults (Keller et al., 2012; Ashburn, 2013). Slacklining is defined as a complex neuromechanical task involving the achievement of functional independence while maintaining dynamic stability. Participants must retain balance while standing or moving on a tightened polyester band (called a slackline) placed between two anchor points at a certain height from the ground (Keller et al., 2012; Pfusterschmied et al., 2013; Gabel, 2014). To maintain balance, the body requires the contribution of neuromuscular, cognitive, and sensory systems (Thomas et al., 2014). This recreational sport has known positive effects on postural stability (Donath et al., 2013) improving balance (Volery et al., 2017; Mildren et al., 2018; Trecroci et al., 2018), neuromuscular control (Jäger et al., 2017), vestibular system function (Dordevic et al., 2017), muscle strength (Granacher et al., 2010), center of gravity control, and posture (Pfusterschmied et al., 2013; Thomas and Kalicinski, 2016). Herold et al. (2017) suggested that challenging balance tasks are primarily related to an increased activity in prefrontal cortex and supplementary motor areas as measured by functional near-infrared spectroscopy. As the execution of slacklining requires coordinating various processes of motor control to maintain balance, it may demand the neural circuitry associated with executive functions (Best, 2010; Diamond and Lee, 2011). Indeed, a study found that acute slickline training increased resting-state functional connectivity between the left lateral prefrontal cortex and the bilateral primary sensorimotor cortex foot area. It suggested that slackline training could be considered a particularly motivating and motor-engaging type of physical exercise that has cognitive benefits. Similarly, Lin et al. (2023) found that school-age children with greater muscular fitness and motor ability (manual dexterity, ball skills, and static and dynamic balance) performed better on visuospatial working memory (VSWM) tasks, highlighting the influence of motor ability on brain health and cognitive development. In addition, Jäger et al. (2014) found that acute coordination exercises have immediate positive effects on inhibition, but not necessarily on updating and shifting. Therefore, the present study aimed to examine the effects of acute slackline exercise on the different aspects of executive functions in a sample of young adults. According to the existing theoretical framework, we expected that acute slackline exercise would improve executive functions in young adults.

## Materials and methods

### Participants

From an initial sample of 58 students in the Department of Sport and Leisure at National Quemoy University participated in this investigation. They were taking a 「Team Building」course and had no prior experience in slacklining. All participants were required to meet the following inclusion criteria: (1) no history of psychiatric or neurological disorders; (2) not taking medicines affecting the central nervous system; (3) normal or corrected-to-normal vision and no color blindness; and (4) no health conditions that could interfere with slacklining.

### Procedure

This study adopted a randomized crossover design, which was approved by the National Taiwan Normal University Center for Research Ethics (202106HS010). Participants first completed screening questionnaires and were then asked about their willingness to participate, and their written informed consent was obtained before participating in the experiment. One week before the intervention, the participants completed a demographic questionnaire and a physical fitness assessment, which included standing long jumps, a sit and reach test, and sit-ups. Their heights and weights were measured using a stadiometer and a digital scale, and waist and hip measurements were made using a measuring tape. Following an explanation of the experimental protocol and its risks, participants provided written informed consent, and then were randomized to different sequences of two sessions, and they all attended the sessions (slackline exercise and film-watching) on two different days separated by at least 24 h. The experimental sessions were counterbalanced in their sequence to minimize any order or learning effects, with half of the participants performing the exercise first and the other half watching the film first. The executive function battery was delivered before and after (10 min) each session (exercise or film). All task instructions were presented to the participants to read, followed by a practice round. Two trained staff members were present in the room for all practices to answer any questions and troubleshoot comprehension issues. The Rating of Perceived Exertion (RPE) assessed using the scale of Borg (1982) was evaluated at the end of each intervention.

Both of exercise and film sessions lasted approximately 50 min. The slackline exercise, consisted of 5 min of warm-up, 15 min of introduction (including setup), and 30 min of the main exercise session in which the participants were instructed to stand on the slackline with one foot and try to keep their balance. If the participants fell off the slackline, they were to stand on the slackline with one foot again within 30 s. If 30 s were exceeded, the teaching assistant reminded the participant to stand on the slackline again. The first goal was stand on the slackline with one foot for 20 s, the second goal was stand on the slackline with one foot for 30 s. In the control session (film-watching), all participants watched a film of a natural landscape from the National Geographic Channel. Watching films is a common sedentary activity and a neutral film was selected to avoid inducing emotional reactions.

### Measures

#### Executive function

Executive function (inhibitory control and cognitive flexibility) was measured using a modified Simon task with three response stages. The target was presented against a black background, and trials began with a white fixation cross. Before the experimental test session, the participants needed to successfully complete a practice session that included six trials. Moreover, they were required to meet a criterion of 100% accuracy in the practice session, which all participants successfully met, before entering the experimental testing session. This requirement was to ensure that the participants comprehended the test. The stimulus remained on the screen for up to 5,000 ms until the participant pressed the left or right bottom of screen, allowing for plenty of time to select the correct response. Participants were requested to respond as quickly and accurately as possible in all stages of the task. If no response was made within 5,000 ms, the trial was recorded as an error. There was an interval of 1,000 ms between each trial. The experiment was designed in three blocks, and there were 136 trials in total. Stage 1 was a neutral familiarization task, followed by 32 trials in which the participants were asked to identify whether a colored circular target that appeared in the center of the tablet screen was red or blue, by pressing on the left bottom for red and right bottom for blue. Stage 2, which measured inhibition capacity, comprised 40 trials with the same rules as Stage 1, but the stimulus could appear on the right or left side of the screen. Thus, this stage contained congruent and incongruent trials. Participants required to resolve stimulus–response conflict arising from incongruent trials in which the stimulus and the response were spatially incompatible (e.g., a stimulus with blue color in the left visual field). The ratio of congruency was equiprobable. Stage 3, which measured cognitive flexibility capacity, contained 64 trials with different rules depending on the shapes of stimulus to demand their cognitive flexibility. That is, participants were required to differentiate between shapes (circles and triangles) and colors (red and blue) of the presented stimulus. They were instructed to press left bottom for a red triangle or blue circle and right bottom for a blue triangle or red circle.

#### Rating of perceived exertion

The participants reported their perception of exertion after each session. Rating of perceived exertion (RPE) was assessed using the scale of Borg (1982), which ranged from 6 to 20. Scores of 6 indicates no exertion at all; scores of 7–11 indicate extremely light to fairly light effort; scores of 12–14 indicate somewhat intense effort; scores of 15–19 indicate intense to extremely intense effort; and a score of 20 indicates exhaustion and maximal exertion.

### Statistical analysis

All measurements were calculated as mean ± standard deviation. Statistical analyses were conducted using SPSS 25 software (IBM Corporation, Armonk, NY, United States). Descriptive data (age, height, weight, body mass index, and waist–hip ratio) were analyzed to summarize the characteristics of the participants. Performance in the modified Simon task was measured as the mean reaction time and the percentage of correct responses (i.e., accuracy). All participants were required to successfully (100% accuracy) complete a practice session to ensure that they understood the requirements for all tasks before taking the experimental test; participants with less than 80% accuracy on the experimental tests were excluded to prevent from inclusion of data from participants who were not focusing on the tests. Furthermore, we trimmed trials that were more than 2.5 SDs from the mean response and only the reaction time data corresponding to correct answers were averaged. The reaction time and accuracy of stage 1 were performed separately using a 2 (intervention: watched film vs. slacklining) × 2 (time: pre-session vs. post-session) repeated-measures analyses of variance (ANOVAs) model. Additionally, the reaction time and accuracy data were separately submitted to 2 (intervention: watched film vs. slacklining) × 2 (time: pre-session vs. post-session) × 2 (condition: congruent vs. incongruent) repeated-measures ANOVAs to examine the effects of the two sessions on stage 2 and 3 data. Findings were reported using the Greenhouse–Geisser correction statistic for violations of sphericity. To estimate the effect size, partial eta-squared was calculated for significant main effects and interactions, and Cohen’s *d* (*d* = t/(√n)) was calculated for *post hoc* comparisons. The level of statistical significance was set at α = 0.05.

## Results

Five participants failed to complete all testing sessions and six participants were excluded because their modified Simon task accuracy was lower than 80%. Accordingly, data from the remaining 47 participants were included in the statistical analysis. The participants’ demographic characteristics are reported in Table 1.

**Table 1:** Characteristics of the participants (*N* = 47).

Characteristics		M ± SD
Gender (Male/Female)		28/19
Age (years)		19.17 ± 1.94
Height (cm)		168.21 ± 7.5
Weight (kg)		61.42 ± 11
BMI (kg/m^2^)		21.64 ± 3.41
WHR		0.8 ± 0.05
Standing long jump (cm)		194.15 ± 34.35
Sit-up (times)		37.91 ± 8.71
Sit and reach (cm)		29.72 ± 11.31
RPE	Film	11.83 ± 3.15
	Slackline	13.11 ± 2.58

M, mean; SD, standard deviation; BMI, body mass index; WHR, waist–hip ratio; RPE, rating of perceived exertion.

### Reaction time

Table 2 shows the participants’ performance of the three stages of the modified Simon task for each intervention. For Stage 1, the results revealed the main effects of TIME, *F*(1,47) = 5.642, *p* = 0.022, η_p_^2^ = 0.109, with post-test exhibiting longer reaction time than pre-test. However, neither the main effect of INTERVENTION, *F*(1,47) = 1.654, *p* = 0.205, η_p_^2^ = 0.35, nor the INTERVENTION × TIME interaction, *F*(1,47) = 3.324, *p* = 0.075, η_p_^2^ = 0.067 were significant.

**Table 2:** Reaction time and accuracy in the modified Simon task for each intervention (M ± SD).

		Reaction time		Accuracy	
		Pre-test (ms)	Post-test (ms)	Pre-test (%)	Post-test (%)
Stage 1	Neutral					Slackline	657.52 ± 58.41	659.25 ± 70.16	98.54 ± 3.21	98.94 ± 2.35	Control (film)	655.26 ± 54.84	677.42 ± 58.21	98.47 ± 2.49	99.2 ± 2.28
Stage 2	Congruent					Slackline	691.31 ± 66.95	670.74 ± 64	98.09 ± 4.07	98.09 ± 3.19	Control (film)	678.91 ± 52.21	688.74 ± 57.32	99.04 ± 2.22	98.19 ± 2.81	Incongruent					Slackline	710.97 ± 61.63	704.91 ± 69.79	97.98 ± 3.21	97.87 ± 3.38	Control (film)	700.31 ± 54.01	717.87 ± 64.11	98.19 ± 3.78	98.4 ± 2.94
Stage 3	Congruent					Slackline	1,055.55 ± 155.9	957.88 ± 119.61	95.48 ± 4.07	96.21 ± 4.22	Control (film)	1,023.02 ± 93.4	951.28 ± 82.88	95.35 ± 4.95	96.61 ± 3.8	Incongruent					Slackline	1,006.35 ± 133.05	925.08 ± 104.43	96.01 ± 3.91	97.8 ± 3.16	Control (film)	981.63 ± 93.88	937.51 ± 81.37	97.01 ± 3.28	97.27 ± 4.21

M, mean; SD, standard deviation.

For Stage 2, the analysis revealed significant main effects of CONDITION, *F*(1,47) = 68.654, *p* < 0.001, η_p_^2^ = 0.599 and interactions between INTERVENTION × TIME, *F*(1,47) = 8.561, *p* = 0.005, η_p_^2^ = 0.157, and TIME × CONDITION, *F*(1,47) = 7.049, *p* = 0.011, η_p_^2^ = 0.133. Decomposition of the INTERVENTION × TIME interaction showed that the post-test reaction time in the slackline session (*M* = 686.92, SD = 62.26 ms) was shorter than in the film-watching session (*M* = 705.23, SD = 55.43 ms), *t*(46) = 2.58, *p* = 0.013, but there was no significant effect between the film-watching and slackline sessions in the pre-test condition (*p* = 0.101). In addition, we found that the post-test reaction time (*M* = 705.23, SD = 56.03 ms) was longer than the pre-test reaction time in the film-watching session (*M* = 689.14, SD = 49.59 ms), *t*(46) = −2.88, *p* = 0.006, whereas no significant difference between pre and post-test in the slackline session was observed (*p* = 0.070). Decomposition of the TIME × CONDITION interaction demonstrated that reaction times for the congruent condition were shorter than for the incongruent condition at pre-test [*t*(46) = −5.47, *p* < 0.001] and post-test conditions [*t*(46) = −8.25, *p* < 0.001]. There were no significant main effects of INTERVENTION, *F*(1,47) = 0.170, *p* = 0.682, η_p_^2^ = 0.004, or TIME, *F*(1,47) = 0.001, *p* = 0.977, η_p_^2^ < 0.001, or INTERVENTION × TIME × CONDITION interaction, *F*(1,47) = 0.871, *p* = 0.356, η_p_^2^ = 0.019.

For Stage 3, the analysis demonstrated significant main effects of TIME, *F*(1,47) = 157.869, *p* < 0.001, η_p_2 = 0.774, CONDITION, *F*(1,47) = 29.969, *p* < 0.001, η_p_^2^ = 0.394, and INTERVENTION × TIME interaction, *F*(1,47) = 4.098, *p* = 0.049, η_p_^2^ = 0.082. Decomposition of the INTERVENTION × TIME interaction only revealed that the post-test reaction time was shorter than the pre-test reaction time in both the film-watching [*t*(46) = 7.64, *p* < 0.001] and the slackline sessions [*t*(46) = 7.962, *p* = < 0.001]. In addition, there was a significant main effect of CONDITION × TIME interaction, *F*(1,47) = 5.431, *p* = 0.024, η_p_^2^ = 0.106. Decomposition of the interaction analysis revealed that reaction time for the congruent condition was shorter than the incongruent condition at pre-test [*t*(46) = −5.33, *p* < 0.001] and post-test condition [*t*(46) = −3.27, *p* = 0.002]. For the congruent condition, pre-test reaction time (*M* = 1039.29, SD = 101.89 ms) was longer than post-test reaction time (*M* = 954.58, SD=87.57 ms), *t*(46) = 10.31, *p* < 0.001. Such effects were also observed for the incongruent condition [*t*(46) = 9.26, *p* < 0.001]. There were no significant effects of INTERVENTION × CONDITION interaction, *F*(1,47) = 3.177, *p* = 0.081, η_p_^2^ = 0.065, and INTERVENTION × TIME × CONDITION interaction, *F*(1,47) = 0.587, *p* = 0.448, η_p_^2^ = 0.013.

### Accuracy

For Stage 1, a repeated-measures ANOVA of accuracy revealed no significant main effect for INTERVENTION, *F*(1,47) = 0.065, *p* = 0.799, η_p_^2^ = 0.001, and TIME, *F*(1,47) = 2.067, *p* = 0.157, η_p_^2^ = 0.043. We also found no interaction effect for INTERVENTION × TIME, *F*(1,47) = 0.23, *p* = 0.634, η_p_^2^ = 0.005.

For Stage 2, a repeated-measures ANOVA of accuracy revealed no significant main effect for INTERVENTION, *F*(1,47) = 1.422, *p* = 0.239, η_p_^2^ = 0.03, TIME, *F*(1,47) = 0.292, *p* = 0.591, η_p_^2^ = 0.006, and CONDITION, *F*(1,47) = 0.952, *p* = 0.334, η_p_^2^ = 0.02. We also found no interaction effect for INTERVENTION × TIME × CONDITION, *F*(1,47) = 1.035, *p* = 0.314, η_p_^2^ = 0.022. Additionally, there was no INTERVENTION × TIME interaction, *F*(1,47) = 0.179, *p* = 0.674, η_p_^2^ = 0.004, INTERVENTION × CONDITION interaction, *F*(1,47) = 0.068, *p* = 0.795, η_p_^2^ = 0.001, or TIME × CONDITION interaction, *F*(1,47) = 0.832, *p* = 0.366, η_p_^2^ = 0.018.

For Stage 3, we found significant main effects for TIME, *F*(1,47) = 0.8.541, *p* = 0.005, η_p_^2^ = 0.157, and CONDITION, *F*(1,47) = 8.881, *p* = 0.005, η_p_^2^ = 0.162, in which the post-test accuracy (97% ± 0.4) was higher than the pre-test accuracy (96% ± 0.4) and the accuracy in the incongruent condition (97% ± 0.3) was higher than that in the congruent condition (95.9% ± 0.4). There were no significant main effect for INTERVENTION [*F*(1,47) = 0.177, *p* = 0.676, η_p_^2^ = 0.004], INTERVENTION × TIME × CONDITION interaction effect, *F*(1,47) = 1.994, *p* = 0.165, η_p_^2^ = 0.042, INTERVENTION × TIME interaction, *F*(1,47) = 0.403, *p* = 0.529, η_p_^2^ = 0.009, INTERVENTION × CONDITION interaction, *F*(1,47) = 0.035, *p* = 0.852, η_p_^2^ = 0.001, or TIME × CONDITION interaction, *F*(1,47) = 0.002, *p* = 0.962, η_p_^2^ < 0.001.

## Discussion

The aim of the present study was to investigate the effects of acute slackline exercise on the executive functions of young adults. Participants showed better inhibitory control performances as indicated by shorter reaction times following acute slackline exercise in comparison to after film-watching. As there was no difference in accuracy between the slackline exercise and film-watching sessions, the shortened reaction time after slackline exercise can argue against a simple speed-accuracy trade-off. Overall, the results suggest that participating in acute slackline exercise results in beneficial effects on executive functions necessitated by inhibitory control relative to film-watching, in college students.

The results of the present study were in accordance with our hypothesis, indicating that college students demonstrate better performance in executive function tasks after slackline exercise in comparison to the film-watching condition. Specifically, compared to the film-watching session, acute slackline exercise generates improvement in inhibitory control condition, whereas no exercise-induced positive effects were found in task conditions (i.e., Stage 1) with less executive control demands. Previous studies have suggested that acute exercise results in selective facilitative effects on cognitive tasks that involve high executive function demands. For example, Chang et al. (2014) found that acute resistance exercise improved the Stroop task of inhibitory control, especially on the task condition necessitating high inhibitory control demands (i.e., Stroop color-word) relative to other conditions that reflect basic information processing (i.e., Stroop neutral conditions). Therefore, the present study supports the selective improvement hypothesis and suggests that exercise with high coordination demands positively affects cognitive tasks requiring inhibitory control processing.

The positive association between the slackline activity and better inhibition in our study may be explained by the activity’s high demand on whole-body coordination and postural control with respect to variable external conditions and their anticipation (Serrien et al., 2017; Mildren et al., 2018). The cognitive stimulation hypothesis postulates that acute exercise improves cognition, with physical activity activating the same brain regions that are used to control higher-order cognitive processes (Best, 2010; Pesce, 2012; Tomporowski et al., 2015). Human brain imaging and animal studies have shown that neuronal structures such as the cerebellum and the frontal lobe are responsible for coordination as well as executive function: the higher the motor demand, the greater the prefrontal cortex activity required during the execution of motor tasks (Serrien et al., 2006). As such, enhanced executive function performance after a single bout of slackline exercise may be attributed to a specific pre- activation of those exact cognitive processes, which are then used in a subsequent cognitive task. Although previous studies have investigated the effects of coordination exercise on cognition in children and adolescents, it is difficult to determine the efficacy of acute coordination exercise on executive functions due to the designed exercise interventions including various combinations of jumping and running activities, which largely necessitated aerobic energy systems. Moreover, our participants were cognitively healthy young adults (mean age = 23.1 years), already functioning near peak cognitive ability in comparison to children or adolescents. The improved inhibition for college adults is important because inhibition has been shown to be closely linked to work productivity. In classrooms and offices, individuals require attention to focus on relevant information while relying on distinct EFs to suppress irrelevant distractions. Given that the vast majority of the extant literature has focused on aerobic exercise or resistance exercise and inhibitory control (Pontifex et al., 2019), the present findings go a step further in understanding the effectiveness of acute bouts of coordination exercise on executive function in young adults. The results provide an alternative exercise perception and have some implications for the everyday lives of college-aged adults (Mischel et al., 2011; Moffitt et al., 2011; Allan et al., 2014).

The post-test reaction time for task requiring inhibitory control was longer than that of the pre-test following the film-watching session, suggesting that a short session of sedentary behavior impaired executive function, which is consistent with Wheeler et al. (2017) finding that sedentary behavior is linked to impaired brain structure and function, and may contribute to cognitive decline and the development of neurodegenerative diseases. Indeed, some studies have shown that acute prolonged uninterrupted sitting can lead to reductions in brain blood flow, cerebral oxygenation, and worse cognitive performances (Miranda Rosenthal et al., 2001; Carter et al., 2018; Burnet et al., 2021). In addition, studies have also found that prolonged uninterrupted sitting is associated with increased levels of fatigue (Dempsey et al., 2018) and decreased plasma levels of norepinephrine (NE) (Wennberg et al., 2016). The activation of the locus coeruleus (LC) and the associated release of NE play important roles in influencing the attention state, which may modulate executive function performance (Aston-Jones and Cohen, 2005; Gelbard-Sagiv et al., 2018). Such arguments may be partly supported by a previous study indicating reductions in P3b amplitude as indexed by the LC-NE system between pre- and posttest in the sitting condition, whereas no changes in P3b amplitude were observed between pre- and posttest in an acute aerobic exercise condition (Pontifex et al., 2015). As such, following a single bout of acute slackline exercise, inhibitory control was only marginally significantly improved (*p* = 0.07) relative to pretest whereas the film-watching resulted in poor inhibitory control performance. Thus, the present study suggests that participating in acute slackline exercise is better than film-watching activities, due to the partial elimination of the detrimental effects of sedentary behavior on cognition.

Both interventions improved the participants’ executive function performance in Stage 3, which targets the cognitive flexibility component of executive function, but no difference between the two interventions was found. This may explain some of our results by task learning effects when the tests were done on the same day. Specifically, in the acute slackline exercise session and film-watching condition, they showed shorter reaction times at post-test than pre-test. This is in line with prior research, Stein et al. (2017) tested the effect of a single bout (a 20-min session) of coordination exercise on children’s performance of executive function tasks and found that children in the intervention condition did not exhibit improved cognitive flexibility relative to children in the comparison condition. Alves et al. (2012) compared the acute effects of aerobic exercise, resistance training, and stretching on inhibitory control and cognitive flexibility in healthy older women. Although aerobic and resistance training sessions both elicited greater improvements in inhibitory control than stretching, changes in cognitive flexibility did not differ across conditions. In contrast, Shukla et al. (2020) found that a 20-min session of moderate intensity aerobic exercise (via cycle ergometer) was effective in improving cognitive flexibility assessed using a task-switching paradigm involving alternating pro- and antisaccades in young adults. Moreover, two studies in adults that reported an effect on cognitive flexibility (Berse et al., 2014; Barenberg et al., 2015) used incremental and intense ergometer cycling (with aerobic and anaerobic demands). Therefore, the effects of acute exercise on cognitive flexibilities was inconsistent and at least some of the contradictory findings may be attributable to differences in the designs of these studies, including differences in settings where the intervention was executed, type of acute physical activity, and examined age groups.

This study has some limitations that warrant caution when interpreting the findings. First, we only examined the effects of 30 min of slackline exercise on cognitive performance. To determine the optimal effects of exercise on cognitive function, different intensities, durations, and modalities of exercise should be simultaneously considered (Chang et al., 2012). Second, the present investigation may have been restricted by measuring the inhibition and cognitive flexibility aspects of executive functions. Chang and Etnier (2009) proposed that the effects of exercise are dependent on different executive function domains and the tasks that measure specific executive function domains. The generalization of the present study’s results and the potential task specificity of the effects should therefore be undertaken with caution. Budde et al. (2008) reported that 10 min of acute bilateral coordination exercises resulted in greater improvement in school children’s concentration and attention than a regular physical education lesson of the same duration. As there was no significant difference in heart rate between the groups in that study, it is possible that the coordination characteristic of the exercises was responsible for the results. Future research should investigate which qualitative exercise characteristics specifically promote executive function and to what extent the effect translates to functional ability. Third, as we did not measure any neural activity in this study, we were not able to elucidate the possible neurophysiological mechanisms underlying the cognitive benefits induced by the acute slackline exercise. Fourth, due to course planning the slackline exercise group and control group, with each condition separated by a period only of 24 h, it needed more time to wash out the training effect. Future studies incorporating brain imaging tools, ideally capable of measuring brain activity during the execution of slacklining tasks, such as wearable non-invasive brain imaging techniques (Seidel-Marzi and Ragert, 2020), should be encouraged. Additionally, slacklining has been compared to watching a film, but there needs to be a comparison with other exercise types to make sure that the coordination demand is driving the change.

## Conclusion

This present proof-of-concept study demonstrated that acute slacklining, a challenging coordination balance exercise, resulted in better inhibitory control in college students, which can benefit conscious focusing and distraction regulation in academic activities.

## Data availability statement

The raw data supporting the conclusions of this article will be made available by the authors, without undue reservation.

## Ethics statement

The studies involving human participants were reviewed and approved by National Taiwan Normal University Center for Research Ethics. The patients/participants provided their written informed consent to participate in this study.

## Author contributions

C-TW and C-LC were responsible for the research idea, implementing the study, and manuscript writing up. H-CC was responsible for guiding in the analysis and interpretation of the data from the executive function task of the findings. C-CL, S-YW, and W-CH were responsible for assisting on the data collection and analysis. C-JH was responsible for the conceptualization, methodology, resources and supervision. T-YC and T-MH were responsible for the conceptualization, discussion of the research idea, supervision of the data collection, editing, and reviewing of the manuscript. All authors contributed to the article and approved the submitted version.

## Conflict of interest

The authors declare that the research was conducted in the absence of any commercial or financial relationships that could be construed as a potential conflict of interest.

## Publisher’s note

All claims expressed in this article are solely those of the authors and do not necessarily represent those of their affiliated organizations, or those of the publisher, the editors and the reviewers. Any product that may be evaluated in this article, or claim that may be made by its manufacturer, is not guaranteed or endorsed by the publisher.
